# Acid Sensing Ion Channels (ASICs) in NS20Y cells – potential role in neuronal differentiation

**DOI:** 10.1186/s13041-016-0249-8

**Published:** 2016-06-24

**Authors:** Zaven O’Bryant, Tiandong Leng, Mingli Liu, Koichi Inoue, Kiara T. Vann, Zhi-gang Xiong

**Affiliations:** Neuroscience Institute, Morehouse School of Medicine, 720 Westview Drive SW, Atlanta, GA 30310-1945 USA

**Keywords:** Acid Sensing Ion Channels (ASICs), Neuronal differentiation, Neurite growth, Dendrites, NS20Y

## Abstract

Cultured neuronal cell lines can express properties of mature neurons if properly differentiated. Although the precise mechanisms underlying neuronal differentiation are not fully understood, the expression and activation of ion channels, particularly those of Ca^2+^-permeable channels, have been suggested to play a role. In this study, we explored the presence and characterized the properties of acid-sensing ion channels (ASICs) in NS20Y cells, a neuronal cell line previously used for the study of neuronal differentiation. In addition, the potential role of ASICs in cell differentiation was explored. Reverse Transcription Polymerase Chain Reaction and Western blot revealed the presence of ASIC1 subunits in these cells. Fast drops of extracellular pH activated transient inward currents which were blocked, in a dose dependent manner, by amiloride, a non-selective ASIC blocker, and by Psalmotoxin-1 (PcTX1), a specific inhibitor for homomeric ASIC1a and heteromeric ASIC1a/2b channels. Incubation of cells with PcTX1 significantly reduced the differentiation of NS20Y cells induced by cpt-cAMP, as evidenced by decreased neurite length, dendritic complexity, decreased expression of functional voltage gated Na^+^ channels. Consistent with ASIC1a inhibition, ASIC1a knockdown with small interference RNA significantly attenuates cpt-cAMP-induced increase of neurite outgrowth. In summary, we described the presence of functional ASICs in NS20Y cells and demonstrate that ASIC1a plays a role in the differentiation of these cells.

## Background

Neuronal differentiation is essential for the development of the nervous system. A hallmark characteristic of differentiation is the sprouting of neurites which later become axons and dendrites. Major changes in membrane proteins are observed during the differentiation, maturation, and development of neurons, for example increased expression of acid-sensing ion channels (ASICs) [1]. Although the precise mechanisms underlying neuronal differentiation are not fully understood, expression and activation of ion channels, particularly those which are Ca^2+^-permeable, have been suggested to play an important role in the process [2–4].

ASICs are proton-gated cation channels belonging to the degenerin/epithelial Na^+^ channel (DEG/ENaC) superfamily. There are at least four genes that encode six alternatively spliced transcripts: ASIC1a, ASIC1b, ASIC2a, ASIC2b, ASIC3 and ASIC4. ASIC1a, a primary subunit highly expressed in the central and peripheral neurons, is highly sensitive to decrease in extracellular pH [5]. Studies using knockout mice have suggested that activation of ASIC1a contributes to synaptic plasticity, learning and memory [6]. It is unclear however whether ASICs play any role in neuronal differentiation. In this study, we first explored the presence and characterized the properties of ASICs in NS20Y cells, a neuronal cell line that has been previously used to study neuronal differentiation. Next, we determined whether ASIC inhibition affects the differentiation of these cells. Our data provides strong evidence that functional ASIC1a channels are expressed in NS20Y cells and that activation of these channels may play a role in neuronal differentiation.

## Methods

### Cell culture

NS20Y cells, derived from the mouse neuroblastoma, were cultured in Dulbecco’s Modified Eagle’s Medium (Invitrogen), supplemented with 10 % FBS, 100 units/ml penicillin, and 100 μg/ml streptomycin. Cells were plated at 10 - 20 % confluence on 35 mm dishes coated with poly-L-ornithine and maintained at 37 °C in a humidified incubator with 5 % CO_2_ - 95 % atmosphere. For differentiation, cells were treated with 1 mM cpt-cAMP for 72 h or 1 mM cpt-cAMP and 10 nM PcTX1 by adding reagents directly to cell media. The culture medium was not changed during the 72 h treatment. The pH during experiments were 7.62 in control, 7.63 in cpt-cAMP, and 7.64 in cpt-cAMP + PcTX1 treated medium.

### Evaluation of neuronal differentiation

Cells in 35 mm dishes were examined at 400X magnification and photographed using phase contrast microscopy (Nikon). Cells were washed three times with extracellular solution (ECF) before photographs were taken. Neurite length and cell complexity were measured using Nikon Image Software (NIS) (Nikon Instruments, Inc., Melville, NY, USA). For each experiment, at least 5 random fields were selected for evaluation. Number of primary dendrites and total neurite length were quantified [7]. In these experiments neurites are defined as any process that extends from the soma. Neurite length (in μm) was quantified by using a free hand line tool measuring the distance from the neurite tip to where the neurite joins the soma [7]. Exclusion criteria included: 1) cell clusters typically greater than or equal to two, 2) where the total neurite length cannot be ascertained because neurites extend out of the field of view, 3) neurites that appear to have formed neurite-neurite or neurite-somatic connections, and 4) cases of extensively branched or overlapped neurites [7].

Sholl analysis is a widely used method in neurobiology to quantify the complexity of dendritic arbors [8]. The Sholl analysis of NS20Y cells was conducted by plotting the number of neurite intersections against the radial distance from the soma [7].

### Electrophysiology

Whole cell patch clamp recordings were performed as described previously [9]. Patch pipettes were pulled by a two-step puller (PP83; Narishige, Tokyo, Japan) from thin walled borosilicate glass (1.5 mm diameter, World Precision Instruments, Sarasota, FL). The pipettes had a resistance of 3–4 MΩ when filled with intracellular solution: 140 mM CsF, 10 mM HEPES, 11 mM EGTA, 2 mM tetraethylammonium chloride, 1 mM CaCl_2_, 2 mM MgCl_2_ and 4 mM MgATP, pH 7.3 (adjusted with CsOH), 290–300 mOsm adjusted with sucrose. Extracellular solution contained: 140 mM NaCl, 5.4 KCl, 2 mM CaCl_2_, 1 mM MgCl_2_, 10 mM HEPES, 10 mM glucose, pH 7.4, 320–330 mOsm. When needed, 300 nM tetrodotoxin was used in ECF to block the Na^+^ currents. PcTX1 (Peptide International) was dissolved in ddH_2_O at 20 μM before adding to extracellular solutions. Amiloride was dissolved in dimethyl sulfoxide (DMSO) at 100 mM before adding to extracellular solutions to obtain final working concentrations. Tetrakis-(2-Pyridylmethyl) ethylenediamine (TPEN) and Zinc Chloride were dissolved in ddH_2_O before adding to extracellular solutions. Unless described otherwise, all chemicals were purchased from Sigma.

Whole cell patch clamp recordings were done with an Axopatch 200B amplifier and Digidata 1320 DAC unit. Unless indicated otherwise, cells were clamped at a holding potential of −60 mV. ASIC currents were elicited by pH drops from 7.4 to acidic pH values as indicated. Currents were activated at least 1 min apart to achieve a complete recovery from desensitization. A multi-barrel perfusion system (SF-77 Warner Instruments, Hamden, CT) was used to achieve a rapid exchange of extracellular solutions. To generate a current–voltage (I–V) relationship, voltage steps were initiated from −100 and +100 mV from a holding potential of −60 mV were applied at 1 s interval. The data were analyzed with pClamp and Sigma Plot software.

### Western blot

Cells were treated with lysis buffer (50 mmol/L Tris–HCl, 150 mmol/L NaCl, 1 % Triton X-100, protease and phosphatase inhibitor cocktail) and collected by scraping into individual aliquots. The samples were put on ice for 30 min, centrifuged at 12,000 g at 4 °C for 30 min, and then supernatants were collected. Protein concentration was measured using the Bio-Rad protein assay kit (Bio-Rad, Hercules, CA, USA). Thereafter, the proteins were mixed with Laemmli sample buffer and boiled at 95 °C for 5 min. Proteins were separated by 10 % SDS-PAGE, followed by electrotransfer to polyvinylidene difluoride membranes. Blots were probed with antibodies against ASIC1 (rabbit anti-mouse/human, 1:1,000; Gift from Dr. Xiangming Zha, University of South Alabama, Mobile, AL, USA) or beta-actin (1:2,000; Abcam, Cambridge, MA, USA), detected using horseradish peroxidase-conjugated secondary antibody (1:1,000; Cell Signaling, Danvers, MA, USA), and visualized by ECL (Amersham Biosciences Piscataway, NJ) and Blue Autoradiography film (MedSupply Partners, Atlanta, GA). The intensity of the protein bands were quantified using NIH Image J software.

### Reverse Transcription – Polymerase Chain Reaction (RT-PCR)

RT-PCR was used to examine the expression of individual ASIC subunits, as described in our previous studies [10]. Total RNAs were isolated from NS20Y cells with Trizol reagent (Invitrogen), according to the manufacturer’s protocol. Equal amount of total RNA was reverse transcribed and PCR amplified with Superscript II (Invitrogen) using specific primers for individual ASIC subunit. ASIC1a forward5′-TCCTATGAGCGGCTGTCTCT-3′, ASIC1a, reverse 5′-TGCTTTTCATCAGCCATCTG-3′, ASIC1b forward 5′-GGCCTTTGTCATAGCACTGGGTGC -3′, ASIC1b reverse 5′-TTCCCATACCGCGTGAAGACCAC -3′, ASIC2a forward 5′-CGCCAACACCTCTACTCTCC-3′, ASIC2a reverse 5′-TGCCATCCTCGCCTGAGTTA-3′, ASIC2b forward 5′-CCTTGGCTTGCTGTTGTCCT-3′ ASIC2b reverse 5′-TGCCATCCTCGCCTGAGTTA-3′, ASIC3 forward 5′-GTCTGGACCCTGCTGAACAT-3′, ASIC3 reverse 5′-GGCTCTGGATCAAAGTCGGG-3′, ASIC4 forward 5′-GGGCTAGCATCCTCACCTTG-3′, ASIC4 reverse (5′-GGCCCAGTTTCATGGGTACT-3′. RT positive (+) samples was run with the reverse transcriptase, while RT negative (−) samples were run without reverse transcriptase. The RT-PCR products were electrophoresed on 1.5 % agarose gel.

### ASIC1a shRNA transfection

Short hairpin ASIC1a (shASIC1a) and control shRNA were purchased from SuperArray Bioscience Corporation (Frederick, MD), each vector contains the shRNA under control of U1 promoter and the GFP gene, for the enrichment of transiently transfected cells. NS20Y cells were transfected with 5 μg of each specific ASIC1a shRNA or control shRNA using Lipofectamine™ reagent in serum free OptiMEM-1 medium (Invitrogen, Carlsbad, CA) per 35 mm dish according to the manufacture’s instruction. After transfection, cells were grown for further 72 h in growth medium as indicated in each experiment before utilization.

### Statistical analysis

Data were expressed as mean ± SEM. Where applicable, multiple groups were compared using analysis of variance (ANOVA). Two groups were compared using Student’s *t*-test (paired and unpaired where appropriate). Values of *p* < 0.05 were considered statistically significant.

## Results

### Detection of ASIC transcript and protein in NS20Y cells

**Fig. 1:**
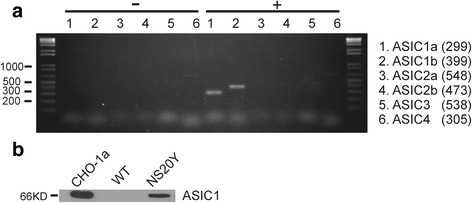
The Expression of ASICs in NS20Y cells. **a** Reverse Transcription (RT) - PCR reveals the presence of ASIC1a and ASIC1b mRNA in NS20Y cells. **b** Western blot reveals the expression of ASIC1a Protein. Chinese Hamster Ovarian (CHO) cells with (CHO-1a) or without (WT) expression of ASIC1a were used as the positive and negative controls

Using RT-PCR, the presence of ASIC transcripts in NS20Y cells was investigated. The mRNA expression of all ASIC subunits including ASIC1a, 1b, 2a, 2b, 3 and 4 were examined. RT-PCR results clearly show the presence of ASIC1a and ASIC1b transcripts at the expected sizes (299 bp and 399 bp) without detection of other subunits (Fig. 1a). We further examined the expression of ASIC1a protein by Western blot. Chinese Hamster Ovarian (CHO) cells and CHO cells with stable ASIC1a expression (CHO-ASIC1a) were used as negative and positive controls. Western blots showed clear immunoreactivity to the ASIC1a in NS20Y cells at the expected molecular weights (Fig. 1b). Taken together, these findings indicate that ASIC1a is expressed in NS20Y cells.

### Characterization of the ASIC currents in NS20Y cells

**Fig. 2:**
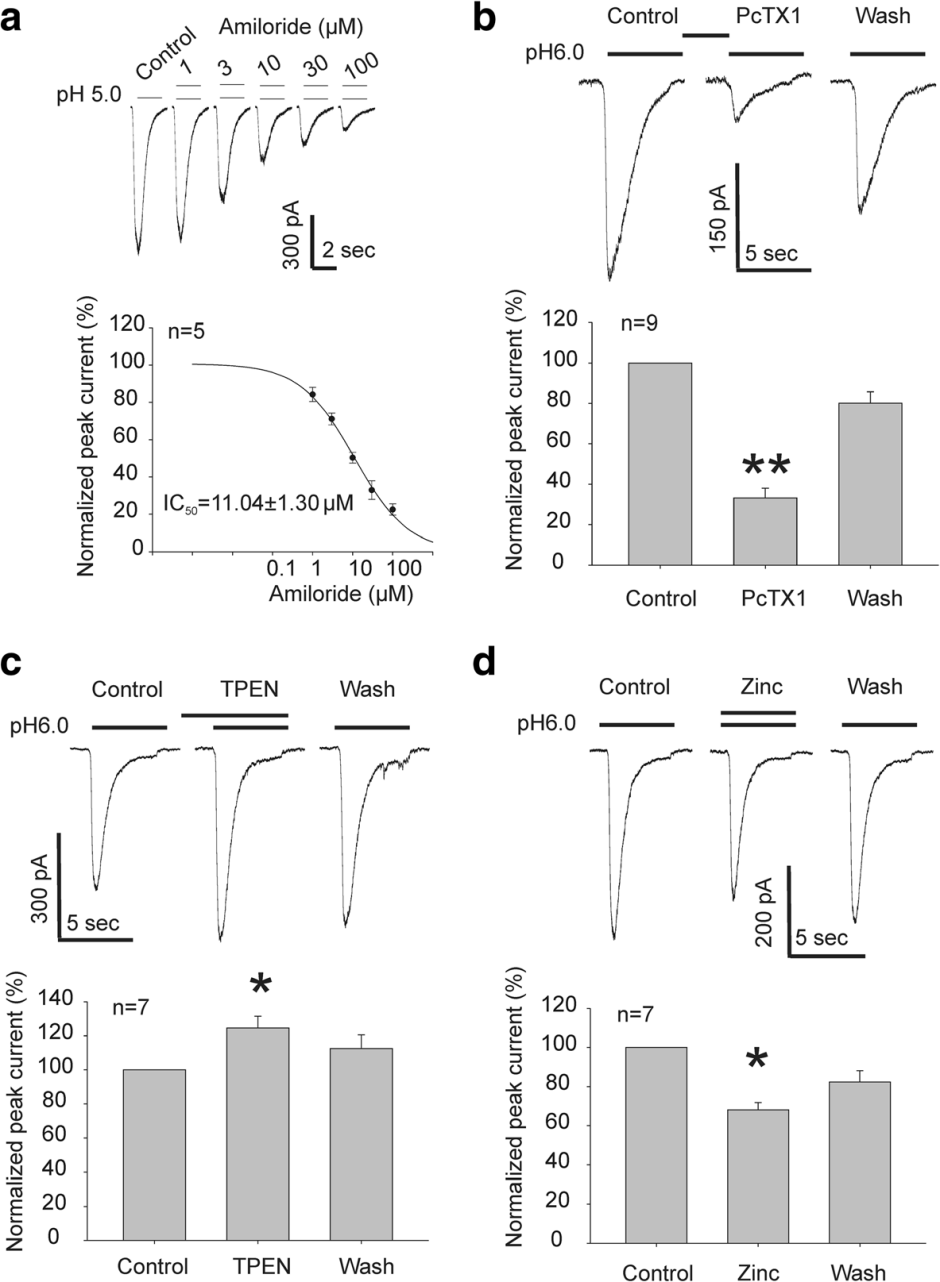
Inhibition of ASIC current by amiloride and PcTX1 and modulation of ASIC current by zinc and zinc chelator in NS20Y cells. **a** Amiloride dose dependently inhibits the ASIC current in NS20Y cells with a half-maximal inhibitory concentration (IC_50_) of 11.04 μM (*n* = 5). **b** PcTX1, at 10 nM, also significantly inhibits the ASIC current in NS20Y cells (**p < 0.01, *n* = 9). **c** Zinc chelator, TPEN, at 100 μM, significantly potentiates ASIC currents (*p* < 0.05, *n* = 7). **d** Zinc, 100 μM causes a significant inhibition of ASIC currents (*p* < 0.05, *n* = 7)

Using patch-clamp recording, we then studied the acid-activated currents and examined the effect of various pharmacologic agents known to modulate ASICs. ASIC currents were induced by an extracellular pH drop from 7.4 to 6.0 [9]. As shown in Fig. 2, drop of extracellular pH from 7.4 to 6.0 induced transient inward currents. Amiloride, a commonly used nonspecific inhibitor of ASICs [11–14], blocked the acid activated currents in NS20Y cells in a dose-dependent manner with a half-maximal inhibitory concentration (IC_50_) of 11.04 μM (*n* = 5) (Fig. 2a).

Psalmotoxin-1 (PcTX1), isolated from the venom of tarantula Psalmopoeus cambridgei, potently and specifically inhibits the proton-gated currents mediated by homomeric ASIC1a expressed in heterologous systems [15]. In addition, PcTX1 also inhibits the current mediated by heteromeric ASIC1a/2b channels [11]. We tested the effect of PcTX1 on ASIC currents in NS20Y cells. As shown in (Fig. 2b), after several minutes of perfusion, PcTX1 (10 nM) produced a significant inhibition of ASIC currents in NS20Y cells (*p* < 0.05, paired student’s *t*-test). In 9 cells tested, an average inhibition of 66.9 ± 14.8 % of the current amplitude was achieved. Since our RT-PCR data did not show clear expression of ASIC2b (Fig. 1), this result suggested that the acid-activated current in NS20Y cells is largely mediated by the homomeric ASIC1a channels.

Zinc modulates ASIC currents, but different ASIC subunit stoichiometry responds differently to zinc; as such, zinc may either potentiate or inhibit the acid-activated currents [16, 17]. For example, at nanomolar concentrations, zinc inhibits ASIC1a containing channels with an IC_50_ of ~10 nM [16]. While at high micromolar concentrations, zinc potentiates ASIC2a containing channels [17]. Zinc chelator tetrakis-(2-Pyridylmethyl) ethylenediamine (TPEN) potentiates ASIC1a current, by removing zinc mediated inhibition. Application of 100 µM TPEN significantly increased the amplitude of ASIC currents in NS20Y cells, further supporting the presence of ASIC1a containing channels (*p* < 0.01, paired student’s *t*-test, *n* = 7, Fig. 2c). We found that addition of 100 μM zinc caused no potentiation but an inhibition of ASIC currents in NS20Y cells (*p* < 0.01, paired Student’s *t*-test, *n* = 7, Fig. 2d), which is consistent with the presence of ASIC1a containing channels.

### ASIC blockade inhibits neuritogenesis

**Fig. 3:**
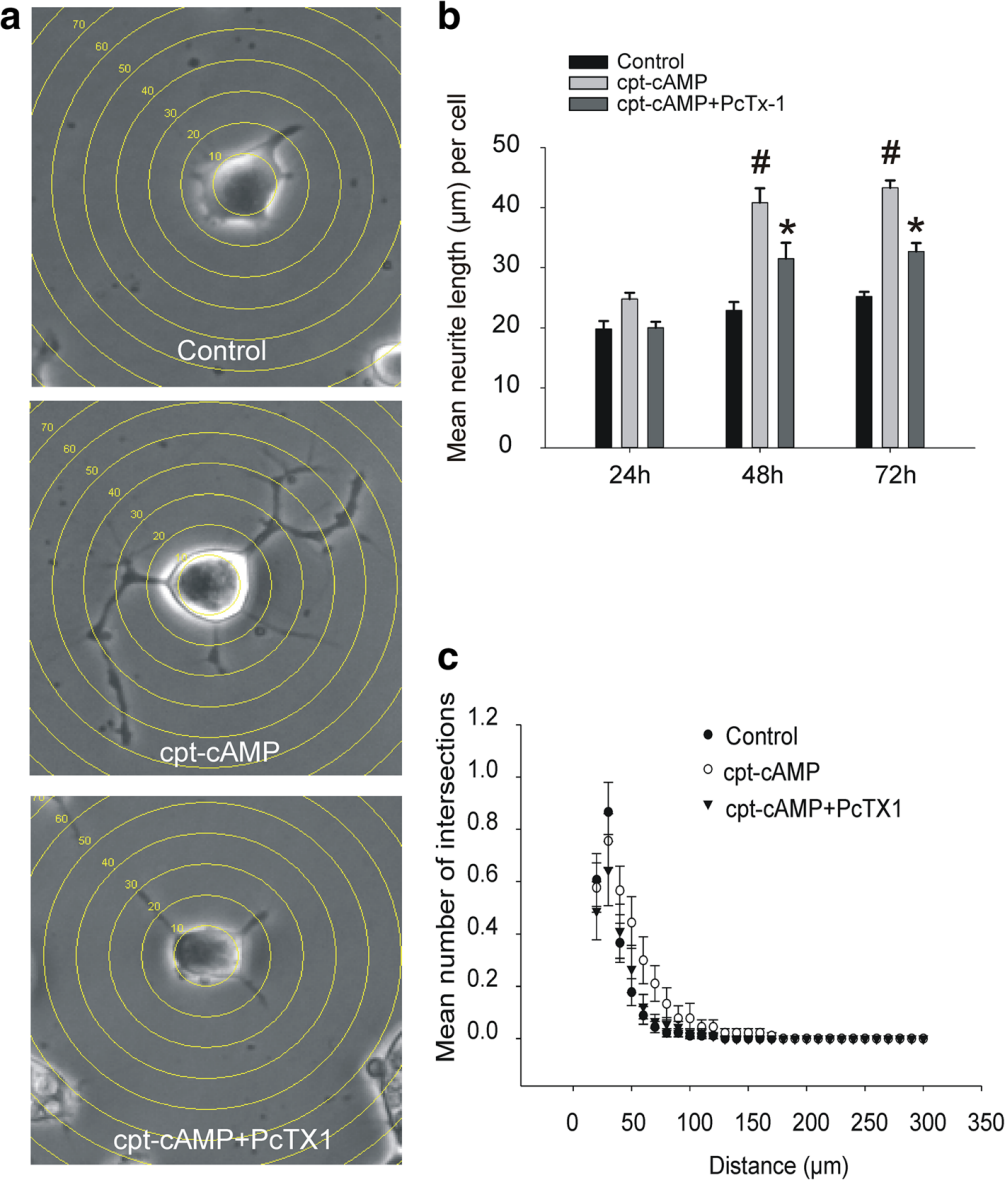
Effect of PcTX1 on cpt-cAMP induced differentiation of NS20Y cells. **a** Example images showing morphology of NS20Y cells in different treatment groups. For Sholl analysis of dendritic complexity, concentric rings were placed at the center of the soma radiating outward at a 10 μm intervals. Neurite complexity is measured by the number of neurites per intersection. Top panel: Control - shows NS20Y cells grown in media without pharmacologic treatment. Cells display a full, rounded soma with few, short undeveloped processes. Middle panel: cpt-cAMP treated - shows NS20Y cells treated with 1 mM cpt-cAMP for 72-h. Cells have large cell bodies, multiple and elongated processes with extensive branching. Lower panel: cpt-cAMP + PcTX1 - shows NS20Y cells treated with 1 mM cpt-cAMP and 10 nM PcTX1 for 72-h. These cells have a reduced neurite extension and processes. Photomicrographs were taken at 400×, phase contrast. **b** Summary data expressed as the mean neurite length/cell at time points of 24, 48, and 72 h. cpt-cAMP significantly increases the mean length of neurite extension (^#^ *p* < 0.05 vs. control). When PcTX1 is added, there is a significant reduction in neurite length compared to cpt-cAMP treated cells (^*^ *p* < 0.05). **c** Summary Sholl analysis data of control, cpt-cAMP (1 mM) treated and cpt-cAMP (1 mM) + PcTX1 (10nM) treated cells at 72 h. The plot shows the mean number of neurite intersections at indicated distance from the soma. Treatment with cpt-cAMP significantly increased the dendritic complexity (*p* < 0.05 vs. control, two way ANOVA, *n* = 90 cells for each group from 3 separate experiments). Co-treatment with 10 nM PcTX1 significantly attenuated the increase of dendritic complexity induced by cpt-cAMP (*p* < 0.05 between cpt-cAMP and cpt-cAMP + PcTX1, two way ANOVA, *n* = 90 cells from 3 separate experiments)

To explore the potential role of ASICs in neuronal differentiation, the effect of ASIC inhibition on cpt-cAMP induced neuritogenesis of NS20Y cells was studied. Treatment of NS20Y cells with 1 mM cpt-cAMP for 72-h has been shown to induce clear differentiation [18, 19]. Differentiation was analyzed morphologically by extension and branching of neurites, and by directly counting the number of primary dendrites (explained in detail in *Methods*). While control cells are lack of extensive branches (Fig. 3a, upper panel), cells treated with 1 mM cpt-cAMP for 72 h show significantly increased neurite number, length, and arborization (Fig. 3a, middle panel), co-incubation of 10 nM PcTX1 with 1 mM cpt-cAMP for 48 and 72 h significantly decreased the number of neurite branching and neurite length compared to 1 mM cpt-cAMP treatment alone (*n* = 264, *p* < 0.05, Fig. 3a, b, c). PcTX1 alone has no significant effect on neurite growth (*p* = 0.38, *n* = 268, data not shown).

The Sholl analysis method has been used extensively in neuronal cultures for the studies of dendritic complexity [20, 21]. We applied this technique in cultures of NS20Y cells (Fig. 3a). Cells treated with 1 mM cpt-cAMP show neurites that significantly extend from the soma and contain more branches than control cells at distances between 50 – 90 μm (*p* < 0.05) (total of 90 cells measured from 3 independent experiments, two-way ANOVA) (Fig. 3c), while cells co-treated with 1 mM cpt-cAMP and 10 nM PcTX1 have neurites that are not significantly different from control (*p* = 0.24) (total of 90 cells measured from 3 independent experiments) at the same distances.

**Fig. 4:**
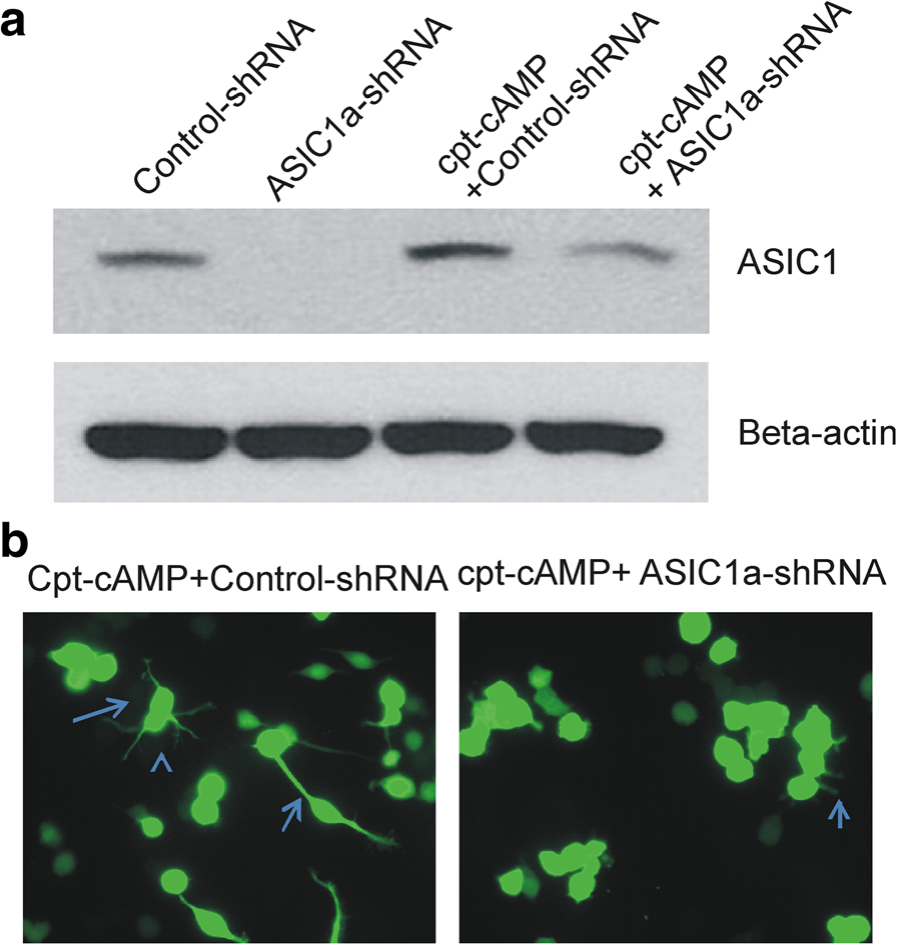
cpt-cAMP mediated neurite extension is reduced by ASIC1a knock-down. **a** Western Blot shows that ASIC1a-shRNA treatment for 72 h significantly decreases ASIC1a expression compared with Control-shRNA treated NS-20Y cells either with or without 1 mM cpt-cAMP treatment. **b** Example photomicrographs of cpt-cAMP treated cells in the presence of control-shRNA or ASIC1a-shRNA as indicated. Blue arrows indicated NS20Y cells with enhanced neurite extension. cpt-cAMP + ASIC1a-shRNA treated cells have less and decreased neurite length indicated by the blue arrow. Increases in neurite length seen with application of 1 mM cpt-cAMP in control-shRNA transfected cells is significantly reduced when NS20Y is transfected with ASIC1a-shRNA (*p* < 0.01) (*n* = 25–44)

PcTX1 is generally accepted to be a specific inhibitor of ASIC1a however there are reports that suggest inhibition of ASIC1a/2b heteromeric channels [11, 15]. To provide additional evidence that ASIC1a is involved in ctp-cAMP-induced differentiation of NS20Y cells, we determined whether knocking down the expression of ASIC1a with small hairpin interference RNA (shRNA) has an effect on ctp-cAMP-mediated neurite extension. After transfection with plasmid containing control-shRNA-GFP or ASIC1a-shRNA-GFP, cells were treated with 1 mM ctp-cAMP for 72 h. Transfection efficiency was confirmed by a decrease of ASIC1a expression as determined by Western blot (Fig. 4a). In cells treated with control shRNA, ctp-cAMP treatment was able to induce clear neurite growth (Fig. 4b), as described above. However, in cells transfected with ASIC1a-shRNA, average neurite length was significantly decreased compared to that in cells transfected with control-shRNA (Fig. 4b, *p* < 0.05, *n* = 25-44).

### ASIC1a expression and current density were increased in differentiated NS20Y cells

**Fig. 5:**
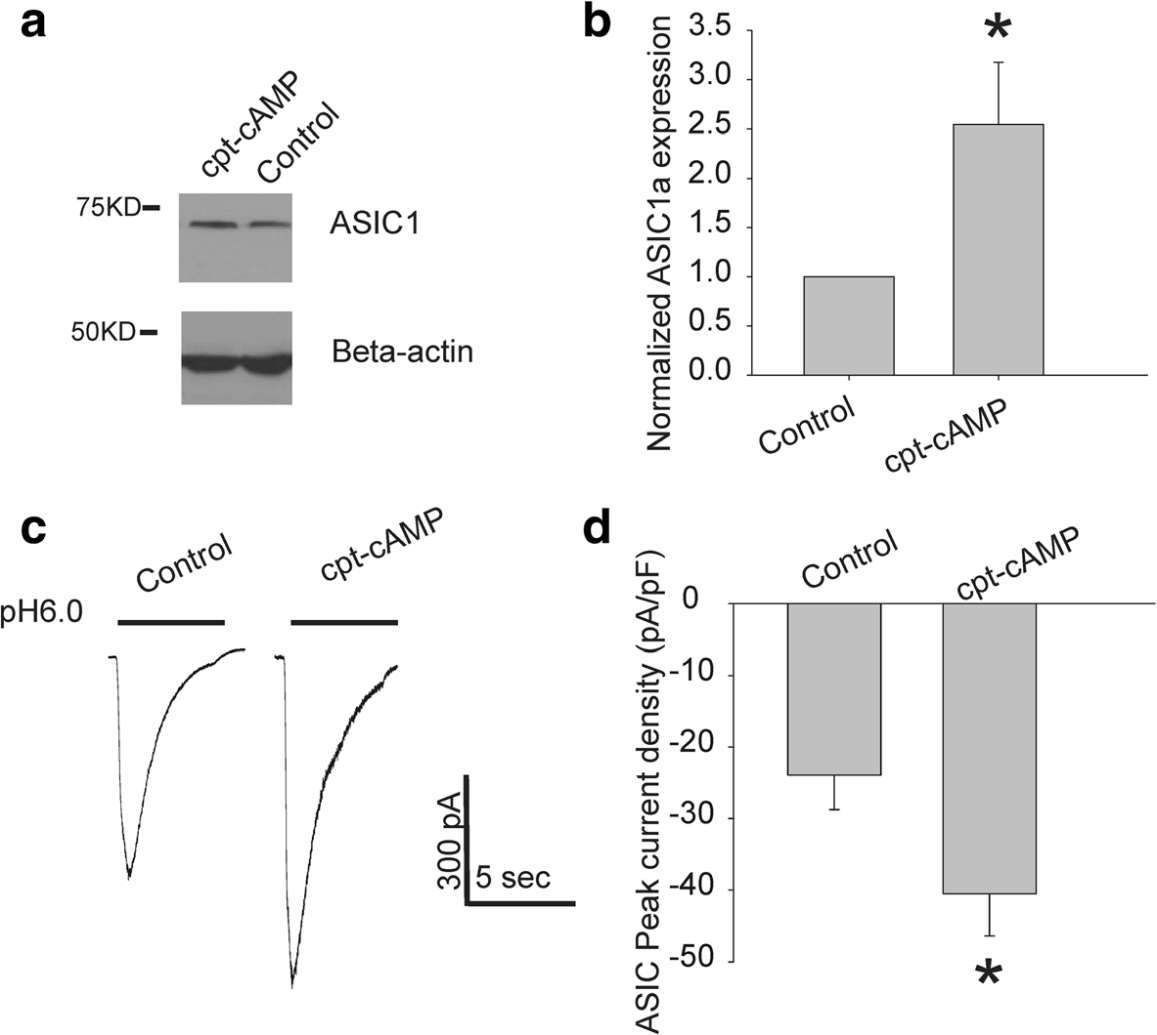
cpt-cAMP increases ASIC1a expression and ASIC1a current. **a** ASIC1a protein expression by Western blot. Blots show immunoreactivity to ASIC1 and Beta-actin at their expected molecular weights. After treatment with 1 mM cpt-cAMP, there is an increase in the expression of ASIC1a as evidenced by an increase in density. **b** Summary data of Western blotting shows that cpt-cAMP significantly increased ASIC1a protein expression (*p* = 0.05) (*n* = 12). **c** Example of whole cell recording that illustrates an increase in ASIC1a current after 72-h treatment with cpt-cAMP. **d** Summary data showing the ASIC current density in NS20Y cells from different treatment groups. There is a significant increase in the density of peak ASIC current in NS20Y cells treated with cpt-cAMP (* *p* < 0.05 vs. control, *n* = 12 in each group)

ASIC protein expression and current density after treatment with cpt-cAMP were also examined. Western blots show an increase in ASIC1a expression after treatment with cpt-cAMP for 72 h (Fig. 5a, b). (*p* < 0.05, *n* = 12). The whole cell patch-clamp recording shows that treatment with 1 mM cpt-cAMP significantly increases the density of ASIC current (*p* < 0.05, *n* = 12, Fig. 5c, d). These findings are consistent with other reports showing that ASIC expression increases with neuronal maturation [1].

### Inhibition of ASIC1a reduces the amplitude of voltage gated Na^+^ current

**Fig. 6:**
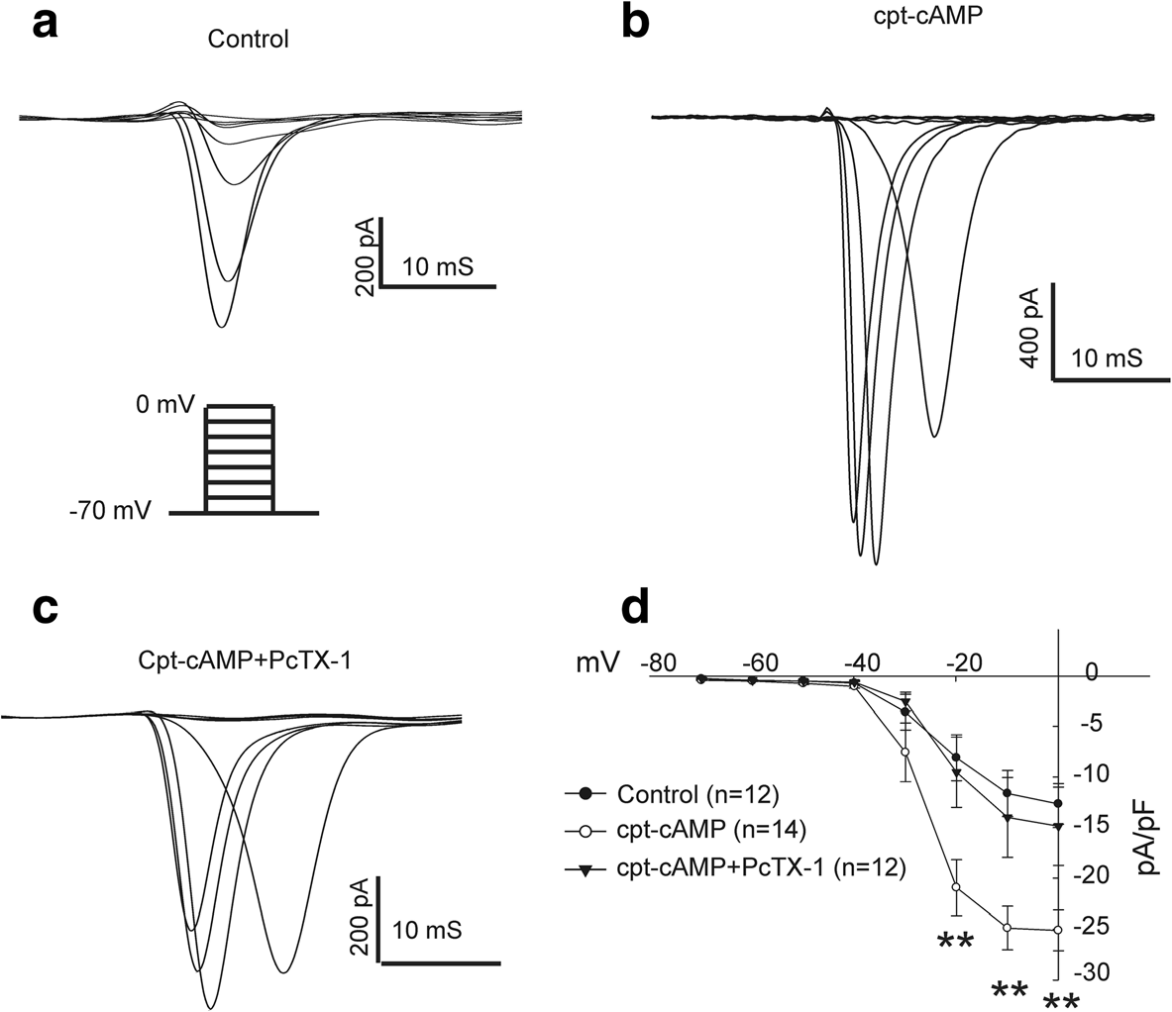
Inhibition of ASIC1a attenuates voltage gated Na^+^ current. **a**, **b**, **c** Example traces showing voltage gated Na^+^ currents in NS20Y cells from different treatment groups. Currents were elicited by voltage steps from −70 mV to 0 mV in 10 mV increments. Treatment of cells with 1 mM cpt-cAMP (**b**) or 1 mM cpt-cAMP + 10 nM PcTX1 (**c**) for 72-h causes a concurrent increase or decrease in Na^+^ peak current amplitude, respectively. **d** Summary I/V plot shows a significant increase in density of voltage gated Na^+^ current after 72-h treatment with 1 mM cpt-cAMP (**p < 0.01), which was largely abolished by PcTX1 (*n* = 12–14)

Voltage gated sodium channels have been known to be exclusive to excitable cells, especially those of neuronal origin [22]. Over the course of neuronal differentiation developing neurons begin to express a wide variety of Na^+^ channels [23]. Here, we found that the amplitude of TTX-sensitive voltage gated Na^+^ current increases with 1 mM cpt-cAMP treatment. The increase of the Na^+^ current by cpt-cAMP is however attenuated by co-treatment with 10 nM PcTX1 (Fig. 6a, b, c, d) (**p < 0.01) (*n* = 12–14 cells). The ratios of cells exhibiting the Na^+^ current are 12/15, 14/14 and 12/14 in control, cpt-cAMP and cpt-cAMP + PcTx-1 treated cells, respectively.

## Discussion

This is the first report, to our knowledge, of the presence of functional ASICs in NS20Y, a mouse neuroblastoma cell line. More importantly, we show that blocking the activity of ASICs inhibits neurite growth/neuronal differentiation. Cyclic-AMP is commonly used to differentiate NS20Y and other clonal cell lines [19, 24]. The use of cyclic-AMPs induces increases in the activities of tyrosine hydroxylase, choline acetyltransferase, the content of poly(A)^+^ cytoplasmic RNA, and causes changes in nuclear non-histone proteins [7, 18, 25]. These molecular changes can be tracked by measuring changes in expression of differentially regulated molecules such as neuropeptides [7]. As expected, treatment with cpt-cAMP resulted in an increased neurite extension, dendritic complexity and increase in Na^+^ current.

To explore a potential role of ASICs in the differentiation of NS20Y cells, we first determined whether NS20Y cells express ASICs. RT-PCR detected the presence of both ASIC1a and ASIC1b transcripts and Western blot confirmed the presence of ASIC1 protein. The presence of ASIC1a subunit was expected as it is fairly ubiquitous in the central and peripheral neuronal tissues [26–28]. While the presence of ASIC1b in NS20Y cells was surprising, it was not unaccounted for as this cell line has heterogeneous origins, composition, and is mainly found in the peripheral nervous system [14, 24].

Acid-sensing ion channels were further characterized using the whole-cell patch clamp technique. In all cells examined, lowering the extracellular pH from 7.4 to pH 6.0 evoked a transient inward current at a holding potential of −60 mV. The properties of acid-activated currents in NS20Y cells resemble ASIC currents in cultured primary CNS neurons (human, mouse, and rat) [9, 29, 30]. For example, ASICs in NS20Y were pharmacologically blocked by the non-specific inhibitor amiloride, and the specific inhibitor PcTX1. The concentration response data of amiloride in NS20Y cells is consistent with previously established IC_50_ for amiloride blockade of ASICs in CNS neurons [9, 30]. In addition, ASIC currents were inhibited by zinc but potentiated by zinc chelation. Zinc inhibition is consistent with the presence of ASIC1a containing channels [16, 17]. It is plausible that homomeric ASIC1b and heteromeric ASIC1a/1b formations may occur, these configurations of ASICs are not sensitive to PcTX1, which is not the case for the current in NS20Y cells where application of PcTX1 inhibited ~70 % of the current. Although PcTX1 also inhibits the current mediated by heteromeric ASIC1a/ASIC2b channels [11, 15], our RT-PCR result did not show clear expression of 2b transcript. Taking together, our data suggest that homomeric ASIC1a channels are predominantly responsible for acid induced currents in NS20Y cells.

Having established the presence of functional ASICs in NS20Y cells, we explored the potential role of these channels in neuronal neuritogenesis. Neuritogenesis was defined as the extension and branching of neurites (length and complexity), similar to other reports in the field [7, 19]. These parameters were quantified by counting the number of neurites/cell and their length from soma to furthest tip. We noted that while control cells remained small and lacked of extensive branches, cells treated with 1 mM cpt-cAMP increased neurite number, length, overall soma size, and arborization. When PcTX1 was used to inhibit the ASIC1a channels cells treated with cpt-cAMP failed to exhibit the same increase of neurite growth. Similarly, knock-down the expression of ASIC1a resulted in a suppression of cpt-cAMP induced neurite extension. Together, these data suggest that ASIC1a may play an important role in neuronal differentiation of NS20Y cells.

ASIC1a has been suggested to play a role in synaptic plasticity, learning and memory [6], and in acidosis-mediated cell death [9, 31]. Since Ca^2+^ permeability/signaling plays a pervasive role in neuronal maturation, dendritic arborization, and axon outgrowth [18, 32, 33], it is plausible that ASIC1a activation may play a role in neuritogenesis and neuronal differentiation. ASICs are spatially distributed and co-localize with postsynaptic density protein-95 (PSD-95) at the soma, along dendritic shafts and spines and importantly at the synapses, suggesting the possible involvement of ASIC in normal synaptic transmission and plasticity [6]. Indeed, when ASICs are removed from the synapse, long term potentiation (LTP), the molecular model for learning memory, is impaired [6]. Previous studies have shown that expression of ASIC1a modulates the density of dendritic spines [34], which may partially explain its role in synaptic plasticity. Our current study suggests that ASIC1a may also play a role in neuronal differentiation and maturation, which may, at least partially, account for its role in synaptic transmission.

NS20Y is a cholinergic cell line which resembles many properties of neurons when differentiated; however, it cannot represent all properties of native neurons and therefore has limitations in neuronal differentiation investigation. Future studies will explore the role of ASIC1a in differentiation/maturation of native neurons.

## Abbreviations

ASICs, acid sensing ion channels; CHO, Chinese Hamster ovarian; CHO-ASIC1a, Chinese Hamster ovarian transfected with ASIC1a; cpt-cAMP, 8 - (4-Chlorophenylthio)-adenosine-3′,5′-cyclic monophosphate, sodium salt; DEG/ENaC, degenerin/epithelial Na^+^ channel; ECF, extracellular fluid; LTP, long term potentiation; PcTX1, psalmotoxin 1; RT-PCR, reverse transcription polymerase chain reaction; TPEN, tetrakis-(2-Pyridylmethyl) ethylenediamine
